# Advances in bacteriophage-mediated strategies for combating polymicrobial biofilms

**DOI:** 10.3389/fmicb.2023.1320345

**Published:** 2024-01-05

**Authors:** Marta Gliźniewicz, Dominika Miłek, Patrycja Olszewska, Artur Czajkowski, Natalia Serwin, Elżbieta Cecerska-Heryć, Barbara Dołęgowska, Bartłomiej Grygorcewicz

**Affiliations:** ^1^Faculty of Pharmacy, Medical Biotechnology and Laboratory Medicine, Pomeranian Medical University in Szczecin, Szczecin, Poland; ^2^Department of Chemical Technology and Engineering, Institute of Chemical Engineering and Environmental Protection Processes, West Pomeranian University of Technology, Szczecin, Poland

**Keywords:** phage therapy, depolymerases, multi-species biofilm, phage-antibiotic synergy, polymicrobial infections

## Abstract

Bacteria and fungi tend to coexist within biofilms instead of in planktonic states. Usually, such communities include cross-kingdom microorganisms, which make them harder to remove from abiotic surfaces or infection sites. Additionally, the produced biofilm matrix protects embedded microorganisms from antibiotics, disinfectants, or the host immune system. Therefore, classic therapies based on antibiotics might be ineffective, especially when multidrug-resistant bacteria are causative factors. The complexities surrounding the eradication of biofilms from diverse surfaces and the human body have spurred the exploration of alternative therapeutic modalities. Among these options, bacteriophages and their enzymatic counterparts have emerged as promising candidates, either employed independently or in synergy with antibiotics and other agents. Phages are natural bacteria killers because of mechanisms of action that differ from antibiotics, phages might answer worldwide problems with bacterial infections. In this review, we report the attempts to use bacteriophages in combating polymicrobial biofilms in *in vitro* studies, using different models, including the therapeutical use of phages. In addition, we sum up the advantages, disadvantages, and perspectives of phage therapy.

## Introduction—biofilm

A biofilm is a structure composed of bacteria and other microorganisms (fungi, viruses) anchored in an extracellular matrix composed of organic substances produced by these microorganisms. Approximately 2–35% of the biofilm’s volume comprises microorganisms, while the matrix constitutes the remaining portion. Biofilm matrix primarily consists of proteins, lipids, polysaccharides, extracellular RNA and DNA, minerals, and ions suspended in water (Vu et al., 2009). Biofilm adheres to the biotic or abiotic surface. The biofilm’s structure may vary on homogeneous, composed of one species, or heterogeneous, consisting of many different strains of microorganisms. Biofilm is more challenging to eradicate than planktonic forms of microorganisms due to the protective properties of the matrix (Augustyniak et al., 2021). The properties of the biofilm enable pathogens to escape from the immune system, antibiotics, disinfectants, and other chemical substances (Jamal et al., 2018; Roy et al., 2018). Microorganisms regulate biofilm formation by expressing genes responsible for synthesizing and modifying extracellular components and communicating with each other by sending biochemical signals. This signaling network includes two-component systems (TCS), which regulate signal transduction via phosphorylation of cyclic di-GMP (c-di-GMP), diguanylate cyclase (DGC) systems which cooperate with TCS and coordinate the transition of bacteria from planktonic to biofilm growth mode, and quorum sensing (QS), mechanism that involves autoinducers which are small signal molecules and receptors (Guła et al., 2018). The signaling occurs interkingdom between microorganisms (bacteria, fungi) and host cells.

### Biofilm formation

The main stages of biofilm formation are initial contact with a surface, irreversible contact with a surface, formation of microcolonies—expansion, maturation of the biofilm, and cell detachment of the individual cells from the matrix. Surfaces susceptible to bacterial adhesion encompass a variety of substrates such as sewage system pipes, soil particles, living tissues, and medical equipment (e.g., urological catheters, venous catheters, artificial heart valves, intrauterine coils, dental units, and contact lenses) (Vu et al., 2009; Stickler, 2014; Augustyniak et al., 2021). Additional cellular structures, such as fimbriae and flagella, bacterial proteins—adhesins, and physical forces, are responsible for the colonization. Environmental factors, such as the amount of available energy, surface structure, pressure, temperature, and orientation of bacterial cells, influence the possibility of adhesion to the substrate. The main physical forces involved in biofilm formation are van der Waals, steric, or electrostatic interactions associated with the cell membrane double layer (Delcaru et al., 2016).

Following the adhesion stage, there is a phase of microbial multiplication, leading to an expansion in the biofilm volume and the formation of a three-dimensional structure regulated by quorum sensing (QS). This mechanism relies on the secretion of proteins and autoinducers of the expression of genes coding for surface proteins, such as porins. This facilitates more effective nutrient absorption within the biofilm. The secretion of exopolysaccharides (EPSs), which stabilize the biofilm structure, also increases. Special channels are created in the entire biofilm volume to facilitate the removal of unnecessary metabolites and provide an appropriate gas environment and nutrients. Due to the static growth, the development of additional membrane structures responsible for the movement of bacteria is inhibited. In addition, a reduction in protease and phospholipase C synthesis, a decrease in the synthesis and release of toxins, and the production of rough and sometimes mucus-like polysaccharides to better adapt cells to specific conditions of the biofilm microenvironment are observed (Jamal et al., 2018; Narayanan et al., 2018; Amankwah et al., 2022).

The final phase of biofilm life occurs when the ratio of newly formed cells equals the number of dead ones. Environmental conditions such as oxygen depletion and nutrient unavailability result in the switching of bacterial metabolism. Enzymes (e.g., hydrolases and endonucleases) that break down the extracellular matrix, allowing individual bacteria to be released into the environment, are produced. In addition, the expression of genes leading to the formation of flagella returns, restoring the ability of bacteria to move and find a new location for biofilm expansion (Garrett et al., 2008).

### Biofilm bacteria virulence and eradication methods

Biofilm production by bacteria is related to their virulence and may imply the occurrence of chronic diseases in the host organism. This is related to many factors, e.g., the production of endotoxins or the protection of bacteria living in the biofilm against the mechanisms of the host immune system, such as phagocytosis or coating with antibodies (Roy et al., 2018). In addition, higher resistance to antibiotics is observed, which is associated with the problematic penetration of active drug ingredients through the biofilm, alternation in biofilm bacteria metabolic activity and presence of cells with a reduced metabolic activity called persister cells, multi-species biofilm, and facilitation of horizontal gene transfer (HGT) among bacteria (Ehrlich et al., 2010; Lehman and Donlan, 2015; Koo et al., 2017).

Biofilm eradication is an enlarging problem in medicine, agriculture, and the food industry. The Center for Disease Control and Prevention (CDC) estimates that even more than 65% of all chronic bacterial infections are caused by biofilm forms of pathogens (Amankwah et al., 2022). One of the novel antimicrobials is lactoferrin, mammalian transferrin with antimicrobial activity, which binds iron, preventing bacteria from using this metal. Another strategy is using molecules that inhibit the mechanisms of the QS system by suppressing signal generation, distribution or blocking signal receptors, and signal responses (Myszka and Czaczyk, 2010). The potential use of substances that influence the structure and work of efflux pumps, which are responsible for removing antibiotics from the bacterial cell, e.g., peptidomimetics, has also been investigated.

The possibility of using phages and phage-derived enzymes to combat bacteria in biofilm structures is also being explored. Furthermore, combination therapy using phages and/or phage-derived products with other antimicrobial agents, including antibiotics, nanoparticles, and antimicrobial peptides, is auspicious. Such a solution could be widely used in medicine to treat severe cases and the broadly understood industry (Herce-Ros et al., 2021; Srinivasan et al., 2021; Tanaka et al., 2021; Amankwah et al., 2022).

## Polymicrobial biofilm

Mixed biofilms occur in many natural environments, e.g., the oral cavity, where many microorganisms form dental plaques, intestines, or vaginas. Certain multi-species biofilm-related diseases can arise when a single pathogen is introduced into an existing microbiome, leading to dysbiosis or when opportunistic pathogens become virulent due to environmental imbalances. Dysbiosis can develop gradually or rapidly and often leads to chronic destructive inflammation. Other situations occur when one pathogen first adheres to the infection site as first and prepares the environment for another. The initial pathogen that adheres to the surface may influence the subsequent bacterial cell selection and, consequently, the final composition of biofilm. It is called coaggregation and may occur when the secondary colonizer binds to specific molecules on the surface of a first one or several bacteria coordinate among themselves and favor some phenotypic changes that lead to the coaggregation on biofilms (Rickard et al., 2003; Peters et al., 2012; Szafrański et al., 2017). Moreover, due to the recruitment of a new species, the gene pool is broadened, and it helps control and regulate the survival mechanisms of individual members, such as adhesion, stimulation of host cellular senescence mechanisms to prevent the shedding of bacteria, and the production of plasma exudate for nutrition through local inflammation (Anju et al., 2022).

The interactions between microbes are complex and involve competition for space and nutrients. The biofilm community’s physiology and function often change and are regulated by various interspecies interactions. Bacterial species are organized into different spatial forms based on their type: interspecific segregation, coaggregation, and stratification (Liu et al., 2016; Anju et al., 2022). Microorganisms grouped in one community may act synergistically, antagonistically, or be indifferent to each other. Cooperation between bacteria facilitates their adhesion and growth of, resistance to antimicrobial agents, virulence, exopolysaccharide production, and protective properties of the whole biofilm.

Moreover, the exchange of nutrients and metabolic products may occur in some species’ relationships. For example, *Fusobacterium nucleatum* and *Prevotella intermedia* produce ammonia, which increases the pH and creates an environment suitable for the growth of *Porphyromonas gingivalis*. Another example is *Pseudomonas aeruginosa*, which produces substances that protect *Staphylococcus aureus* from aminoglycosides (Wolcott et al., 2013; Anju et al., 2022). The opposite behavior is observed when antagonistic interaction occurs. Then, one microorganism inhibits or kills the competing species, ensuring itself to avail available space, energy sources, and nutrients. Competition can be exploitative and involves the superiority of energy utilization or interference that produces compounds preventing other species’ growth (Mgomi et al., 2022).

Multi-species biofilms can also be characterized by the distribution of microorganisms within the matrix. Microorganisms may coexist in separate microcolonies, with limited interactions, in one style of organization. Another style is characterized by a thoroughly mixed arrangement where cells from different species randomly coexist throughout the biofilm. One species forms the bottom layer in the third organizational structure, while the second species places on top (Mgomi et al., 2022). Another scheme is frequently observed in bacteria–fungi biofilms where hyphae form a scaffold that carries bacteria cells (Bernard et al., 2020; Roszak et al., 2022). Different structures of biofilms generate different interactions between species and mechanisms of cellular responses for therapies.

Biofilm-related chronic infection is frequently polymicrobial. Coexisting in multi-species communities increases genetic material exchange between cells, metabolic cooperation, development of antibiotic resistance, niche optimization, host immune system modulation, and virulence induction (Kifelew et al., 2019; Mgomi et al., 2022). Creating a standard matrix on tissues or medical devices is a characteristic of population virulence, making the behavior of polymicrobial societies distinct from mono-species. These societies can alter their physical properties in response to the environment and evolve through mutation to better adapt to their surroundings (Ehrlich et al., 2010). Moreover, additional pathogens can be integrated into the biofilm, and the primary ones can mutate to improve the interaction with other resident species, producing a more stable and productive community. All these properties cause more severe disease symptoms than mono-species infections.

One of the biggest problems associated with polymicrobial infection is increased resistance to antimicrobial agents, which might be higher than in mono-species biofilm. It is caused by the extensive diversity of EPSs produced by heterogeneously distributed bacteria that disturb drug penetration (Topka-bielecka et al., 2021). Moreover, some bacteria and fungi can produce polysaccharides or other substances that protect themselves or cells of partner species from antibiotics and antifungal agents. Another threat is interspecies HGT, which results in gene exchange between evolutionarily distant species. This may create bacteria and fungi with different phenotypes with new features that may increase their virulence and drug resistance.

Biofilm-associated polymicrobial communities are responsible for many diseases, e.g., bone infections and osteomyelitis, gall bladder disease, various chronic middle-ear disease processes, and chronic rhinosinusitis, chronic infections of the urogenital systems, e.g., bacterial vaginosis, dental infections, tonsillitis, surgical site infections, chronic non-healing wounds such as venous and diabetic ulcers, pressure sores, and burn injuries, respiratory infections, e.g., cystic fibrosis and medical device-related infection (Ehrlich et al., 2010; Peters et al., 2012; Szafrański et al., 2017; Iszatt et al., 2021; Uyttebroek et al., 2021). Some of these diseases were subjected to phage therapy. In addition, many scientists investigated various possibilities for phage treatment in *in vitro* research.

## Bacteriophages and mechanisms of biofilm combating

Bacteriophages (phages) are viruses that infect bacteria and cannot multiply outside their host cells. Phages were discovered independently by Frederick Twort and Felix d’Hèrelle over 100 years ago and are the most diverse and numerous life forms on the earth. They show high host specificity, recognizing their host at the species and even strain level due to presence of characteristic receptors on the surface of the bacterial cell (Drulis-Kawa et al., 2015; Atshan et al., 2023). The use of bacteriophages is extensive. It covers many areas of life, e.g., medicine and veterinary (phage therapy), food industry (disinfectants of surfaces), agriculture (plant growth promoters), biotechnology and pharmacy (nanocarriers of drugs, biosensors, or diagnostic molecules), and diagnostic (phage typing) (Cowley et al., 2015; Drulis-Kawa et al., 2015). Currently, phages are classified by the European Union (EU) as medical products and by the Food and Drug Administration (FDA) as drugs. Good manufacturing practice (GMP) must be implemented during phage particle production. Nevertheless, all clinical trials are conducted as a therapy of a last chance according to Article 37 of the Declaration of Helsinki and need the permission of the ethical commission. The preparation of consistent legislation regarding the usage of phages in medicine is still ongoing (Patey et al., 2019).

The rate at which bacteria acquire antibiotic resistance is alarming, and the current epidemiological situation requires the search for alternative methods of combating bacterial infections. One of the options is to use bacteriophages (Iszatt et al., 2021). The usage of phages has many advantages, e.g., rapid clearance from organisms, self-propagation in the site of infection, host specificity, opportunity to make a genetic modification, easy isolation, stability, and relatively low-cost production (Łubowska et al., 2019; Mgomi et al., 2022). The right phage must be selected carefully because not all have good therapeutic results. When choosing phages, some rules should be followed: specificity to target bacteria, lytic activity, and the lack of genes encoding bacterial virulence factors, antibiotic resistance products, and toxins. Only fully sequenced bacteriophages can be used for treatment in medicine. Another concern about phage therapy is to optimize the dosage of virions and the method of administration to provide good delivery to the site of infection (Morrisette et al., 2019). The pharmacokinetics of phages are complicated due to their ability to self-replicate. After killing all pathogenic bacteria, the phages are removed from the body as they cannot multiply in eukaryotic cells. In addition, if selected carefully, phages are safe for the human microbiome (Sartini et al., 2021). On the other hand, they may be neutralized by the host’s immune system, removed from the body too quickly, and bring no profit. Another issue from the immune system might be an allergic reaction that may limit the scope of possible use of bacteriophages. Unfortunately, bacteria have developed multiple resistance mechanisms to phages (e.g., modification and blockage phage receptors on the bacterial surface), and even though phages have an equally impressive assortment of tools to overcome this resistance, it is better to use a cocktail of phages (Chegini et al., 2021). Notably, phage resistance observed *in vitro* may not necessarily translate to *in vivo* conditions. This discrepancy arises from the fact that the most prevalent resistance mechanisms often involve alterations in the cell surface that untenable host infection by the phage (Park et al., 2014; Iszatt et al., 2021). Another issue is that phage therapy could release bacterial endotoxins during bacterial cell lysis, which occurs as an effect of phage infection.

Next, to phages themselves, lytic enzymes that they produce are also considered as treatment factors. Bacteriophages synthesize enzymes such as peptidoglycan hydrolases, holins, and endolysins, which allow to release progeny virions by destroying bacterial cells walls (Sousa et al., 2023). Based on their mechanism of action, we can divide them into hydrolases and lyases. Both groups can degrade polysaccharides, including capsular polysaccharides (CPSs), lipopolysaccharides (LPSs), O-polysaccharides, or exopolysaccharides (EPSs), and sometimes polypeptides and lipids (Topka-bielecka et al., 2021). Endolysin can induce lysis from within as an antimicrobial agent active against pathogens. This refers mainly to Gram-negative bacteria, which need to treat with additional factors, e.g., holins that allow the endolysin to move through the cytoplasmic outer membrane and reach the peptidoglycan layer (Mgomi et al., 2022). Other phage enzymes—depolimerases, can be tail-spike proteins with the enzymatic domain or occurring as free molecules. Phage-delivered enzymes are usually unique and species-specific. However, sometimes enzymes can show activity across a broad spectrum between strains and species (Chegini et al., 2021). Treatment of polymicrobial communities usually requires several different enzymes or combined therapy. Bacteria rarely evolve resistance to lysins because they attack sites on the peptidoglycan cell wall critical to bacterial viability. Nevertheless, combining phage lysins and antibiotics, phages and other agents, or the production of genetically engineered enzymes may be more effective in infection elimination.

Lytic phage can be an effective weapon in the fight against biofilm, both in the context of preventing its formation and its eradication. The attempts to use phages as prevention factors include coating urinary catheters and disinfectants in hospital or industry environments (Curtin and Donlan, 2006; Lehman and Donlan, 2015; Melo et al., 2016; Santiago and Donlan, 2020). The activity of phages in mature biofilm depends on the bacteria growth phase, placement, coaggregation with other cells, nutrient availability, access to receptors, and diffusion capacity. Phages can be used not only in biofilms of multi-bacteria species but also in bacteria–yeast ones. *P. aeruginosa* infecting phage Pf4 can inhibit *Candida albicans* biofilm formation, possibly by sequestrating iron (Nazik et al., 2017; Pohl, 2022). Phages act differently from antibiotics. They produce enzymes, e.g., depolymerases (DP), that can destroy biofilm matrices made of polysaccharides, including EPS or alginase, breaking down the alginate matrix produced by *P. aeruginosa* (Peters et al., 2012; Santiago and Donlan, 2020). Moreover, phages can stimulate the host bacteria to produce EPS-degrading enzymes and proteases that degrade bacterial capsules. Phages oppositely to antibiotics can degrade bacteria that manifest low metabolic activity due to nutrient depletion. Another mechanism that helps overcome the matrix is to diffuse through water channels or to adsorb to motile bacteria and “have a ride” to the target site (Kifelew et al., 2019; Amankwah et al., 2022; Atshan et al., 2023). These actions collectively enhance the effectiveness of phages in combating biofilms. Initially, they facilitate the penetration of phages, allowing entry into the biofilm for subsequent replication within bacterial cells. The elevated bacterial density within biofilms significantly amplifies phage infection, leading to the release of new virions. Even when targeting cells with reduced metabolic activity, lytic phages prove effective by releasing intracellular materials. This release stimulates bacterial metabolism, ensuring sustained efficacy (Amankwah et al., 2022). Basting an expanded host range, polyvalent phages emerge as valuable assets in disrupting polymicrobial biofilms.

Phages also exhibit adaptability during isolation, potentially enriched by employing multiple bacterial hosts rather than a singular one. Additionally, an alternative strategy involves leveraging phages as quorum quenchers. Some phages eliminate bacteria conventionally and produce enzymes that disrupt bacterial signal molecules, providing a multifaceted approach to biofilm intervention (Kifelew et al., 2019; Santiago and Donlan, 2020).

Microbial communities have mechanisms of protection from phages that affect phage ability to adsorb, penetrate, diffuse, and proliferate in biofilm. The ability of biofilm to resist phage invasion depends on its age, shape, structure, and morphology. Bacteria can evolve to be insensitive to phage by changing their phenotypes in response to heterogeneous environments*. P. aeruginosa* may transform into a pili-defective variant to avoid infection of phages that use these structures as their receptors (Yamamoto et al., 2021). Biofilm matrix comprises many bacterial enzymes, e.g., amidases and peptidases, that may inactivate phages. Moreover, in deeper biofilm layers, more dead cells occur, and phages may adsorb to them without any benefits for therapy. Molecules can also catch virions in the matrix (Pires et al., n.d.). One of the ways of bacteria defense is the production of systems that interfere with phage nucleic acids, e.g., clustered regularly interspaced short palindromic repeats (CRISPR)–Cas9 (Yang et al., 2020). Bacteria may also prevent phage DNA integration by a superinfection exclusion system or use an abortive infection system to block the synthesis of phage particle compounds (Pires et al., n.d.). Another protection is related to hiding binding phage receptors by the production of curli polymer (CsgA) as extracellular fibers that curtain bacterial cells (Vidakovic et al., 2017; Santiago and Donlan, 2020).

To intensify the action of phages, they can also be used with various groups of antibiotics (phage-antibiotic synergy (PAS) phenomenon). However, not every drug acts synergistically with selected phages and every combination should be checked in *in vitro* studies. For example, the synergistic effect may arise from the stimulation of lytic phage development in the presence of beta-lactam antibiotics. Bacteria under beta-lactam stress change their morphology, facilitating phage assembly and increasing bacterial sensitivity to phage lysins (Comeau et al., 2007; Chegini et al., 2021). Another mode of action of phages is to interfere with bacterial efflux pumps, which increases the sensitivity to various drugs (Chan et al., 2016). Bacteriophages can also be combined with disinfectants such as chloride, hypochlorite, or quaternary ammonium compounds and enzymes, e.g., polysaccharides depolymerases. In addition, in the case of a biofilm with a heterogeneous structure, it is possible to use a phage cocktail composed of several bacteriophages showing bacteriolytic activity against various bacterial pathogens (Comeau et al., 2007). Another alternative to enhance phage penetration through the matrix is debriding biofilm mechanically before phage treatment (Pires et al., n.d.).

**Figure 1:**
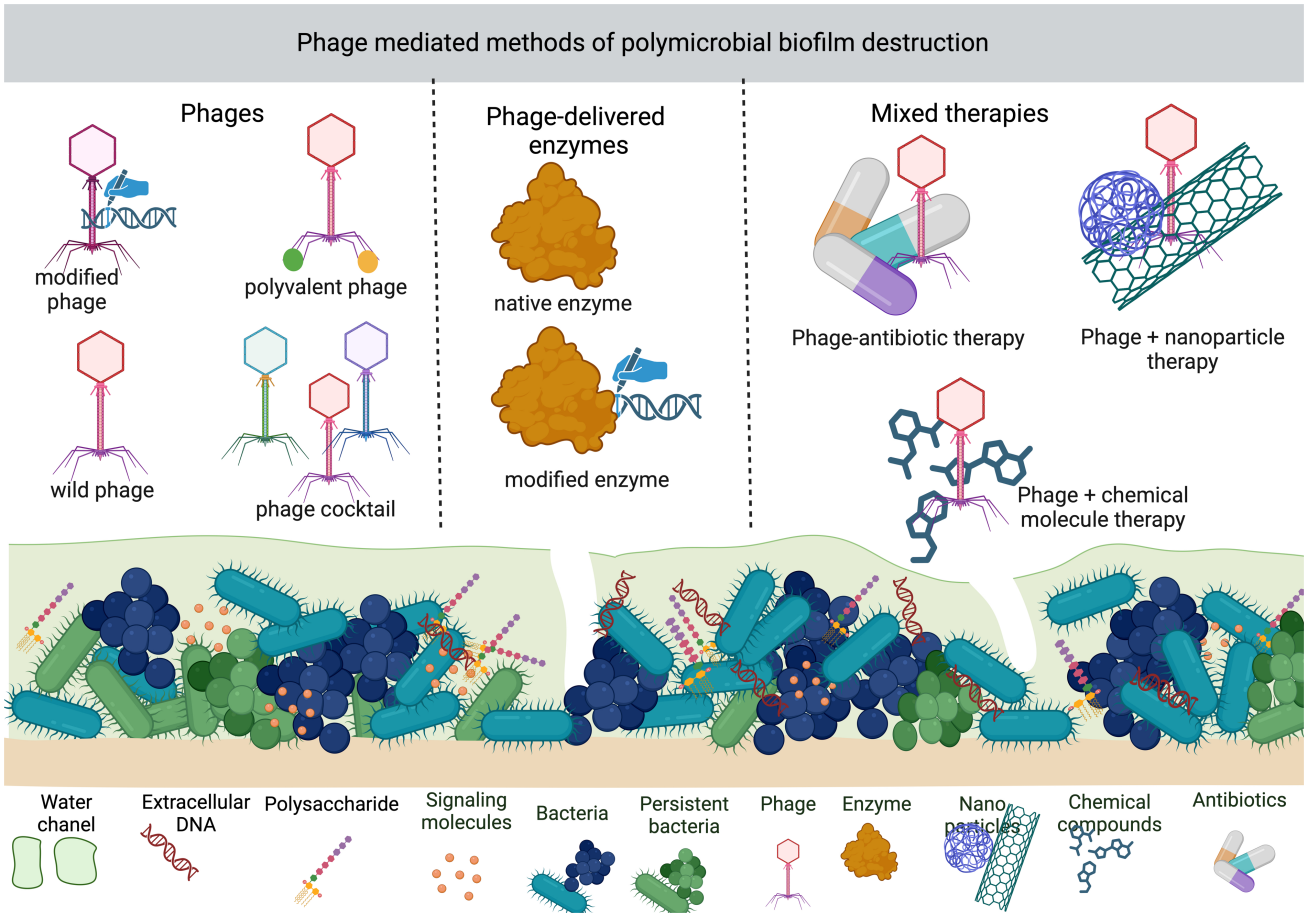
Methods of disturbing polymicrobial biofilm by phage-mediated methods.

Another way to improve phage performance is to modify their genome or synthesize novel ones (Javed et al., 2019). Modified phages may have inserted gene coding for additional exopolysaccharide-degrading enzymes for better biofilm penetration. Bacteriostatic phages can be changed to suppress the DNA repair mechanism, or overexpression of sensitizing proteins, and to disrupt the cell–cell communication between the bacteria in the biofilm. Another target for phage engineering is to use phages as a modulators of antibiotic resistance or to make it possible to reach intracellularly bacteria present in eukaryotic cells (Hagens et al., 2004; Lu and Collins, 2007, 2009; Edgar et al., 2012; Pei and Lamas-Samanamud, 2014). Since sometimes bacterial lysis leads to release toxins and pro-inflammatory products, phages can be engineered to be toxic for bacteria but not lytic for their host (Szafrański et al., 2017). The possibilities of degrading polymicrobial biofilm using phage-mediated methods are presented in Figure 1.

### Bacteriophages as a component of multi-species communities

Bacteriophages should be recognized as a potent tool against pathogenic bacteria and integral components of healthy microbiomes, including those in the oral, intestinal, or vaginal environments. Phages interact with commensal bacteria, fungi, and chemical compounds and contribute to microbial communities assembly, stability, and function. They contribute to biofilm formation as extracellular DNA release through phage-mediated cell lysis may induce mobile genetic element transfer between microbes which, in turn, triggers a response of stabilizing the biofilm matrix (Amankwah et al., 2022). Some phages and their hosts developed reciprocal predator–prey relationships, e.g., in the intestine, phages may promote the evolution of bacterial resistance to phages in response to infection (Duerkop, 2018). Furthermore, phages may bind to mucin glycoproteins, providing phage-mediated antibacterial protection of animal mucosal surfaces (Barr et al., 2013).

Bacterial and phage composition in the intestine depends on diet and may drastically change during infection or other diseases. Increased or changed phage dsDNA levels were noticed during inflammatory bowel disease and type 1 diabetes in children (Zhao et al., 2017; Duerkop, 2018). Phages may stably multiplicate in their host for weeks but not lead to the elimination of pathogens. For example, enteroaggregative *Escherichia coli* (EAEC) and its phages may coexist without resolution, linked to persistent colonization and prolonged diarrhea (Maura et al., 2012). Beyond the intestine, in different niches, the phageome (bacteriophage community in the niche) of the bacterially infected site may be distinct from the healthy one, e.g., during cystic fibrosis (*CF*) (Reece et al., 2021). Phages, as a component of the polymicrobial community, may cooperate with the mammalian immune system and actively eliminate bacteria from the lungs during infection. Oppositely, *P. aeruginosa* prophages can stabilize biofilm in the lungs by promoting attachment to lung mucus and restricting the dispersal of cells from the biofilm. Moreover, phages may decrease the pro-inflammatory response of the immune system and lead to chronic infection (Duerkop, 2018). In another niche, lysogenic phages may modulate the number of vaginal lactobacilli during bacterial vaginosis (Jung et al., 2017). In addition, the taxonomic composition of phages may change during infection, e.g., chronic wound virome is more diverse than contralateral skin, which may influence microbial community and impact healing outcomes (Verbanic et al., 2022).

## Elimination of pathogens from polymicrobial biofilm by phages

Numerous researchers explore phage therapy as a potential solution in the era of limited options for treating antibiotic-resistant bacterial infections. Various approaches are investigated, for example, using phages to eradicate mono-and multi-species biofilm, prevent biofilm formation, or change the composition of multi-species biofilm by removing only the harmful species. The *in vitro* studies implementing phage therapy and the research results employing various models are summed up in Table 1.

**Table 1:** Examples of phage treatment of polymicrobial biofilms in *in vitro* and *in vivo* studies with models.

No.	Pathogens	Aim of the study	Phages used	Experimental model	Outcome	References
1.	*S. aureus* IPLA16/*Lactobacillus plantarum* 55-1 or *Lactobacillus pentosus* A1 and B1 or *Enterococcus faecium* MMRA	Elimination of *S. aureus* from dual-species biofilm	Phage phiIPLA-RODI against *S. aureus*	biofilm formation in 96-well plates for 5 or 24 h at 32°C or 37°C, then phage treatment: 10^7^, 10^8^, or 10^9^ PFU/well for 4 h in nutrient limitation conditions; BIOFILM formation for 5 h, then phage treatment 10^6^ or 10^9^ PFU/well for 18 h in nutrient-rich conditions	5-h treatment with 10^9^ PFU/Well Preparation: Decreased the biomass of *S. aureus*-*L. plantarum* and *S. aureus*-*E. faecium* biofilms by 31 and 67%, respectively In nutrient limitation conditions, *S. aureus* cell counts were reduced by 0.8 and 0.7 log_10_ units 24-h treatment with 10^9^ PFU/Well Preparation: Resulted in an 18 and 63% decrease in the biomass of *S. aureus*-*L. plantarum* and *S. aureus*-*E. faecium* biofilms, respectively In nutrient limitation conditions, 0.4 and 0.6 log_10_ units reduced *S. aureus* cell counts Effects on *S. aureus*-*L. plantarum* Biofilm Biomass: After treatment with a 10^9^ PFU/well preparation, the biomass increased by 120% Viable cell counts for *S. aureus* decreased by 2.0 log_10_ units, while counts for *L. plantarum* increased by about 2.3 log_10_ units in nutrient-rich conditions Effects on *S. aureus*-*L. pentosus* A1 and *S. aureus*-*L. pentosus* B1 Biofilms: The biomass of *S. aureus*-*L. pentosus* A1 biofilm decreased by 86% Cell counts of *S. aureus* decreased by 2.9 and 1.8 log_10_ units after treatment with 10^9^ and 10^6^ PFU/well preparations, respectively No significant difference in *S. aureus*-*L. pentosus* B1 biofilm biomass, but cell counts of *S. aureus* decreased by 1.7 and 0.7 log_10_ units after treatment with 10^9^ and 10^6^ PFU/well preparations, respectively, in nutrient-rich conditions Phage Treatment Observations: In all biofilms treated with a 10^6^ PFU/well preparation, there were increases in phage particles, signifying phage multiplication Conversely, those treated with a 10^9^ PFU/well preparation exhibited no alterations in the number of viable phages	González et al. (2017)
2.	*S. aureus* IPLA1-rifR/*Staphylococcus epidermidis* LO5081	Dual-species biofilm eradication	Phage (phiIPLA-RODI) against *S. aureus* and phage (phiIPLA-C1C) against *S. epidermidis*	biofilm formation in 96-well plates for 24 h at 37°C, then phage treatment: 10^9^ PFU/well separately or together for 4 h	phiIPLA-RODI Treatment: Reduced *S. aureus* by 4.27 log_10_ units Reduced *S. epidermidis* by 2.66 log_10_ units phiIPLA-C1C Treatment: Reduced *S. aureus* by 3.23 log_10_ units Reduced *S. epidermidis* by 2.64 log_10_ units Mixture of Phages: The combined use of phages did not enhance the bacterial count reduction compared to individual phages Application of both phages resulted in higher reduction in biofilm biomass compared to individual phage treatments	Gutiérrez et al. (2015)
3.	*E. coli* MG1655/*P. aeruginosa* PAO1	Dual-species biofilm eradication	Phage λW60 (ATCC 97537) against *E. coli* and phage PB-1 (ATCC 15692-B3) against *P. aeruginosa*	biofilm formation on silicone rubber disks placed in flasks with LB medium and inoculated with *E. coli* and *P. aeruginosa* 10^6^/mL for 2 days at 37°C with shaking, then phage treatment (MOI = 10) for 5 days with daily media refreshment	*E. coli* and *P. aeruginosa* Levels in Biofilm: Regardless of the presence of one or both phages, levels of *E. coli* and *P. aeruginosa* in the biofilm remained relatively constant Phage Resistance Development: *E. coli* demonstrated less resistance to its corresponding phage compared to *P. aeruginosa*	Kay et al. (2011)
4.	*S. aureus* KUB7/*P. aeruginosa* PAO1	Dual-species biofilm eradication	Phage cocktail AB-SA01 (J-Sa-36, Sa-83, Sa-87) against *S. aureus*; Phage cocktail AB-PA01 (Pa-193, Pa-204, Pa-222, Pa-223) against *P. aeruginosa*	biofilm formation in 96-well plates for 48 h at 37°C with shaking, then phage treatment: AB-SA01 9.1 log_10_ PFU/mL and AB-PA01 10.3 log_10_ PFU/mL	Cell Reduction in Biofilm: Treatment resulted in a similar reduction in cell numbers for both *S. aureus* and *P. aeruginosa* compared to individual phage cocktails Specific Reduction Levels: When only AB-SA01 was applied: 1.6 log_10_ PFU/mL When AB-SA01 + AB-PA01 were applied together: 1.2 log_10_ PFU/mL When only AB-PA01 was applied: 2.5 log_10_ PFU/mL When AB-SA01 + AB-PA01 were applied together: 2.1 log_10_ PFU/mL	Kifelew et al. (2020)
5.	*P. aeruginosa* clinical isolates/ *Proteus mirabilis* clinical isolates	biofilm formation prevention on urinary catheter	Phage cocktail (φPaer4, φPaer14, M4, 109, φE2005-A, φE2005-C,) against *P. aeruginosa*; phage cocktail (φPmir1, φPmir32, φPmir34, φPmir37) against *P. mirabilis*	Flowing catheter reactor model. Hydrogel-coated Foley catheters were pretreated with one or both cocktails (*P. aeruginosa* phages 10^9^ PFU/mL; *P. mirabilis* phages 3 × 10^8^ PFU/mL) for 1 h and challenged with 10^3^ CFU/mL of bacteria pumped through the catheters at 1 mL/ min for 2 h in artificial urine medium, then sterile medium was pumped through the catheters at 0.5 mL/min for up to 4 days	Effects of phage pretreatment on *P. aeruginosa* biofilm counts: Phage pretreatment resulted in a reduction of *P. aeruginosa* biofilm counts by 4 log_10_CFU/cm^2^ over 24 h and 48 h The population was eliminated by 72 h, irrespective of the continued presence of phages Effects of phage pretreatment on *P. mirabilis* biofilm counts Phage pretreatment led to a reduction of *P. mirabilis* biofilm counts by 2 log_10_ CFU/cm^2^ over 24 h and 48 h The population continued to decline by 72 h, regardless of the presence of phages	Lehman and Donlan (2015)
6.	*E. coli* HU2117/*P. aeruginosa* EAMS2005-A	biofilm formation prevention on urinary catheter by *P. aeruginosa*	Phage φE2005-A against *P. aeruginosa*	Silicone catheter segments were exposed to *E. coli* 10^5^ CFU/mL and phage 10^8^ PFU/mL for 24 h at 37°C with shaking, then inoculated with *P. aeruginosa* 10^5^ CFU/mL for 30 min and transferred to new flask with human urine for 24, 48, or 72 h at 37°C with shaking	Adherence Reduction in 24 h Experiments: *P. aeruginosa* adherence to catheters was almost 4 log_10_ units lower when pretreated with *E. coli* and phage compared to no pretreatment Adherence Reduction in 72 h Experiments: *P. aeruginosa* adherence to catheters was more than 3 log_10_ units lower with pretreatment compared to no pretreatment Isolated *P. aeruginosa* Counts from Pretreated Catheters: *P. aeruginosa* isolated from *E. coli* and phage-pretreated catheters was 3.1 log_10_ units lower at 24 h, 4.8 log_10_ units lower at 48 h, and 4.5 log_10_ units lower at 72 h compared to untreated catheters *P. aeruginosa* was completely eradicated from catheters in eight out of 27 (30%) experiments when catheters had been pretreated with *E. coli* and phage	Liao et al. (2012)
7.	*S. aureus* Rumba – bovine mastitis isolate/*E. coli* KKH 001 – clinical isolate	Dual-species biofilm dispersal	Phage φ44AHJD against *S. aureus* and phage ɸX174 against *E. coli*	biofilm formation on glass covers for 96 h with daily media refreshment at 37°C with shaking, bacteria inoculum 10^8^ CFU/mL; then phages treatment 10^8^ PFU/mL (one or both phages) for 96 h at 37°C with shaking	Untreated Control: The biofilm intensity of the untreated control consistently decreased over a period of 192 h Phage ɸ44AHJD Treatment: Initially, no visual difference in biofilm intensity was observed until 72 h Subsequently, an increase of 26% in biofilm intensity was noticed after 96 h Phage ɸX174 Treatment: No visual difference in biofilm intensity was seen until 48 h An increase of 28 and 39% in biofilm intensity was noticed after 72 h and 96 h, respectively Combined Phage Treatment (ɸX174 and ɸ44AHJD): No visual difference in biofilm intensity was observed Biofilm intensity decreased to 6% after 96 h	Manoharadas et al. (2021)
8.	*E. coli* CECT 434 and CECT 515/*Salmonella Enteritidis* Ex2 and 269	Dual-species biofilm formation control	Phage Daica against *E. coli;* phage ɸ135 against *Salmonella*	biofilm formation in 96-well plates for 24 h at 37°C with shaking, then phage treatment: MOI = 1 for 24 h at 37°C with shaking	*E. coli* 434 + *Salmonella Enteritidis* Ex2 Biofilm: Reached the lowest numbers of viable cells at 8 h of treatment *E. coli* 434 reduction: 1.15 Log_10_ *Salmonella Enteritidis* Ex2 reduction: 0.88 Log_10_ *E. coli* 515 + *Salmonella Enteritidis* 269 Biofilm: Reached the lowest numbers of viable cells at 4 h of treatment *E. coli* 515 reduction: 1.07 Log_10_ *Salmonella Enteritidis* 269 reduction: 2.42 Log_10_ at 8 h	Milho et al. (2019)
9.	*P. aeruginosa* PAO1/*E. coli* BL21 and TG1	biofilm formation prevention	Engineered T7 phage incorporating the acyl homoserine lactones AHL aiiA gene from *Bacillus anthracis* degraded AHLs	biofilm formation in 96-well plates, inoculated total number of CFU for the mixture of *P. aeruginosa* PAO1, *E. coli* TG1, and *E. coli* BL21 was 5 × 10^7^, with phage (T7wt or T7aiiA) 10^4^ PFU/mL for 24 h at 37°C	Reductions in Biofilm: T7aiiA phage caused reductions of the biofilm by 74.9 and 65.9% at 4 and 8 h post-plating, respectively T7wt phage caused reductions of 23.8 and 31.7% at 4 and 8 h, respectively, compared to the no-phage control Cell Counts at 8 h: At 8 h, the control biofilm reached an average cell count per well of 8.5 × 10^8^ CFU T7wt-treated biofilm had an average cell count of 4.1 × 10^7^ CFU T7aiiA-treated biofilm had an average cell count of 1.2 × 10^7^ CFU PFU Counts in Biofilm: PFU counts for T7wt and T7aiiA in the biofilm were 4.6 × 10^5^ and 4.8 × 10^5^ PFU, respectively	Pei and Lamas-Samanamud (2014)
10.	*P. aeruginosa* ATCC 10145 and *P. aeruginosa* PA01/*C. albicans* CECT 1472	Elimination of *P. aeruginosa* from dual-species biofilm	Phage ɸ IBB-PAA2 and phage ɸ BB-PAP21 against *P. aeruginosa*	biofilm formation in 24-well plates, inoculation 1.9 × 10^9^ CFU/mL for *P. aeruginosa* ATCC 10145 or 1.1 × 10^9^ CFU/mL for *P. aeruginosa* PAO1 and 1.1 × 10^7^ CFU/mL for *C. albicans* for 24 h with media refreshment every 12 h at 37°C with shaking, then phage treatment (MOI = 1) for 24 h at 37°C with shaking	*P. aeruginosa* Inhibition of *C. albicans*: *P. aeruginosa* caused inhibition of the proliferation of *C. albicans* in mixed biofilm without phage Phage Treatment on *P. aeruginosa*: Both phiIBB-PAA2 and phiIBB-PAP21 phages achieved a 2.0 and 1.5 log_10_ reduction, respectively, in the number of viable cells of *P. aeruginosa* 6 h post-infection *P. aeruginosa* Viability at 24 h post-infection: At 24 h post-infection, an increase in the number of viable cells of *P. aeruginosa* was noticed The increase was 1.5 log_10_ for *P. aeruginosa* ATCC 10145 strain and 1 log_10_ for *P. aeruginosa* PAO1 compared to the CFU numbers 6 h post-infection *C. albicans* CFU Increase: An increase of 0.5 and 1 log_10_ in the CFU of *C. albicans* was observed in the presence of *P. aeruginosa* PAO1 and ATCC 10145, respectively, at the 24 h time point	Pires et al. (2013)
11.	*Pseudomonas fluorescens* PF7 and/*Staphylococcus lentus* SL58	Dual-species biofilm eradication	Polyvalent phage ɸ IBB-SL58B against *S. lentus*, T7-like phage (phage ɸ IBB-PF7A) against *Pseudomonas*	biofilm formation on stainless steel slide for 72 h at 30°C with media refreshment every 12 h with or without shaking, then phage treatment of both or only phage ɸIBB-PF7A (both: 10^7^ PFU/ mL)	Dynamic Conditions: The phage cocktail significantly reduced the 72-h-old biofilm by 4 orders of magnitude Phages demonstrated high efficiency in disrupting biofilm structure under dynamic conditions Static Conditions: Phages showed less efficiency in destroying biofilm under static conditions, with only a 10-fold decrease observed after 4 h of phage treatment Viable Cell Release: Phage application to the biofilm induced the release of viable cells (103 CFU/mL) into the planktonic phase Phage Replication in Dual Species Biofilm: Both phages, fIBB-SL58B and fIBB-PF7A, replicated well in the dual-species biofilm Infection with the *Pseudomonas* phage alone resulted in a 100-fold increase in the number of *S. lentus* cells in the planktonic phase compared to biofilm treatments with a cocktail of phages	Sillankorva et al. (2010)
12.	*Enterobacter cloacae* NCTC 5920/*Enterobacter agglomerans* industrial surface isolate *(Ent)*	Dual-species biofilm eradication	Phage ɸ1.15, 11,229 and Blackburn against *Enterobacter cloacae* NCTC 5920 and Philipstown phage against *Enterobacter agglomerans* strain *Ent*	biofilm formation on glass coverslips for 16 h at 30°C, then phage treatment of one or various phage cocktails (MOI = 0.1, 0.01, and 0.001) for 24 h	When Phage ɸ 1.15 was added, there was a reduction of the susceptible strain by 4.0, 3.7, and 4.75 log_10_ CFU/cm^2^ when MOI = 0.1, 0.01, and 0.001 were applied, respectively. The reduction of the unsusceptible strain was 3.2, 3.7, and 0.75 log_10_ CFU/cm^2^ when MOI = 0.1, 0.01, and 0.001 were applied, respectively When Phage Philipstown was added, the reduction of the susceptible strain was 2.9, 2.3, and 3.1 log_10_ CFU/cm2 when MOI = 0.1, 0.01, and 0.001 were applied, respectively. The reduction of the unsusceptible strain was 3.0, 0.4, and 0.5 log_10_ CFU/cm^2^ for MOI = 0.1, 0.01, and 0.001 applied, respectively When Phages ɸ 1.15 and 11,229 were added, there was a reduction of the susceptible strain by 5.0 log_10_ CFU/cm^2^ when MOI = 0.01 was applied, and the reduction of the unsusceptible strain was 2.2 log_10_ CFU/cm^2^ when MOI = 0.01 was applied When Phages ɸ 1.15, 11,229, and Blackburn were added, there was a reduction of the susceptible strain by 5.7 log10 CFU/cm2 when MOI = 0.01 was applied, and the reduction of the unsusceptible strain was 2.1 log_10_ CFU/cm^2^ when MOI = 0.01 was applied (all data read from the original figures)	Tait et al. (2002)
13.	*P. aeruginosa* PAO1/*P. aeruginosa* PA14	Elimination by the phage of the sensitive strain from dual-species biofilm	Phage 352 against PAO1,	biofilm was formed as colony onto agar on membrane filter for 12 h at 37°C (PAO1 10^4^ CFU/mL and PA14 10^5^ or 10^6^ CFU/mL) then filter was transferred to new plate with drop of phage 10^6^ or 10^9^ PFU/ mL then incubated for 36 h at 37°C	PAO1 population size was reduced in the phage treated mixed colonies. Microscopy revealed the absence of PAO1 cells from the edges of the colonies treated with phages, suggests that cell lysis occurred at the actively growing edges and not in the middle of the colony Coculture colonies contained a lower infectious load (fewer phage per sensitive bacteria) compared to mono-culture colonies at the end of the experiment; phage could replicate less in the presence of PA14 Phage resistance was much less likely to emerge in mixed colonies	Testa et al. (2019)
14.	*Cupriavidus metallidurans* 101480065–2, *Chryseobacterium gleum* 113330055–2, *Ralstonia insidiosa* 130770013–1, *Methylorubrum populi* 122620021–1, *Sphingomonas paucimobilis* 121220007–2, *Ralstonia pickettii* 113330051–2	Prevention of *S. paucimobilis* presence in multi-species biofilm; elimination of *S. paucimobilis* from multi-species biofilm	Phage ɸ Scott against *S. paucimobilis*	biofilm formation in 96-well plates for 24-96 h at 30°C, no shaking, then phage treatment with 2 × 10^4^ PFU/mL either at 0 h, or 24 h post-inoculation	The application of bacteriophage ɸScott at the beginning resulted in the absence of *S. paucimobilis* at 24 h of biofilm formation in mixed cultures Phage treatment of pre-existing BIOFILM resulted in no substantial biofilm removal – 20–50 CFU reduction for *S. paucimobilis*	Thompson et al. (2020)
15.	*E. coli* AR3110/*Vibrio cholerae* N16961 (serogroup O1 El Tor)	Elimination of *E. coli* from dual-species biofilm	Recombinant T7 phages against *E. coli*	biofilm formation of *V. cholerae* and *E. coli* into the microfluidic chambers bonded to glass coverslips at a ratio of 2:1 for 48 h and then treated with phages 5 × 10^6^ PFU/μL for 16 – 96 h	After phage introduction, most *E. coli* cells lysed. Over the next 16 h, *E. coli* cells embedded on the bottom layers of *V. cholerae*-dominated cell groups largely survived phage exposure. Persisted *E. coli* was observed up to 144 h but did not appear to be active After 16 h in the dual species biofilm, T7 infection could be seen proceeding partially into groups of *E. coli* embedded within *V. cholerae* biofilm, but a fraction of *E. coli* survived	Winans et al. (2022)
16.	*E. faecalis* Efa1/*E. faecium* C410	Dual-species biofilm eradication	Phage vB_EfaS-Zip against *E. faecium* and vB_EfaP-Max against *E. faecalis*	biofilm formation on collagen wound model (CWM) in 24-well plates for 48 h at 37°C with shaking, with daily media refreshment; then phage treatment 10^8^ PFU/mL of each phage for 24 h	Cell concentration was reduced by approx. 2.5 log CFU/mL after 3 h of infection, however phage resistance occurred and after 24 h of phage infection the reduction was only of 1.0 log_10_ CFU/mL	Melo et al. (2019)
17.	*S. typhimurium* ATCC 14028 and *E. coli* O157: H7	Dual-species biofilm eradication; biofilm formation prevention	Polyvalent phage STP55 against multiple serotypes of *Salmonella* and *E. coli*	biofilm formation in 96-well plate (for prevention): both bacteria inoculum 10^9^ CFU/mL, phage concentration 10^8^ PFU/mL, incubation for 6, 12, and 24 h at 37°C; (for eradication): both bacteria inoculum 10^9^ CFU/mL, incubation at 37°C for 24 h, then phage treatment 10^8^ PFU/mL, incubation at 37°C for 2, 6, and 8 h Spiked lettuce model: lettuce pieces were submerged in bacterial suspension (10^9^ CFU/mL) for 2 min then dried and incubated for 24 h at 37°C, then phage treatment 10^8^ PFU/mL for 10 min, dried and incubated for 2 h at 37°C	Prevention: the increase in the biomass of biofilm was suppressed in the presence of phage. After 6 h it was 48.6% lower and after 24 h it was 52.8% lower than in the control; cells count was lower than those of the control by 1.7, 1.1, and 1.3 log_10_CFU/well, respectively, at 6, 12, and 24 h Eradication: More than 46.2% of the biofilm was removed after 8 h of phage treatment Spiked lettuce model: After phage treatment, the structure of the biofilm changed: net-like matrix had a much flatter and looser structure, the dense structures were dispersed, and the matrix richness of the mixed cells was reduced, the dense structures were dispersed, and the matrix richness of the mixed cells was reduced	Zhu et al. (2022).
18.	*E. coli* K-12 (ATCC 700926)/*Pseudomonas. putida* F1 (ATCC 700007)/*Bacillus subtilis* 168 (ATCC 23857)	Elimination of *E. coli* from the multi-species biofilm	Polyvalent phage Pef1 against *E. coli* and *P. putida* or coliphage T4 against *E. coli*	biofilm formation in glass vials filled with quartz sand, each bacteria inoculum 10^5^ CFU/mL, incubation for 24 h at 30°C with shaking, then media refreshment with bacteria and phage T4 or Pef1 at 10^6^ PFU/mL, incubation for 5 days with daily media refreshment	Pef1 was 20-fold more effective than T4 in suppressing *E. coli*, *E. coli* concentration was 1.3 orders of magnitude lower (4.7 log_10_CFU/mL) than in microcosms with T4 after 3 days in the presence of Pef1; Pef1 proliferated better than T4 After Pef1 amendment, the density of the attached 5-day-old *E. coli* biofilm decreased by 93% to 4.51 log_10_ CFU/mg sand, with T4 it increased by 44% to 5.80 log_10_ CFU/mg sand	Yu et al. (2017)
19.	KPC+ *Klebsiella pneumoniae* (CAV1016)/*P. aeruginosa*, *Micrococcus luteus*, *Stenotrophomonas*. *Maltophilia*, *Elizabethkingia nopheles*, *Cupriavidus metallidurans*, and *Methylobacterium fujisawaense*	*K. pneumoniae* elimination from multi-species biofilm	Phage cocktail (SNP1_2017, SNP2_2017, SNP3_2017, RLS1_2017) against *K. pneumoniae*	biofilm formation on CDC biofilm reactor (CBR) p-trap model for 28 days; phage treatment for 2 h at either 25°C or 37°C with the phage cocktail (10^9^ PFU/mL) at 7, 14, and 21 day post-inoculation	Phage treatment reduced *K. pneumoniae* viability by 1 log_10_ CFU/cm2 at 7 and 14 days (37°C) and 1.4 log_10_ and 1.6 log10 CFU/cm2 at 7 and 14 days, respectively (25°C), no significant reduction was observed at 21 day post-inoculation. Phage treatment had no significant effect on the biofilm heterotrophic plate counts at any time point or temperature. Supplementation with a non-ionic surfactant appears to enhance phage association within biofilm	Santiago et al. (2020)

## Mixed therapies based on phages or phage-delivered enzymes

Even though phages occurred to be effective to some point in biofilm-forming prevention and eradication of mature polymicrobial biofilm, some limitation of phage therapy must be overcome to achieve fully effectiveness. The main problems are acquiring phage resistance by bacteria in polymicrobial biofilm, reaching target bacteria in this complex structure, or selecting phages for all pathogens in biofilm. The solution might be combined therapy based on phages mixed with antibiotics, nanoparticles, other substances, or using phage-delivered enzymes with different properties than phages.

Different approaches to using phage-antibiotic synergy (PAS) therapy are listed in Table 2. The selection of good phage-antibiotic pair is strictly individual to the bacterial strain and case (Grygorcewicz et al., 2023). However, the effort is worth it and brings better results than monotherapy. Phage may prevent the development of antibiotic-resistant minority bacterial populations, and conversely, antibiotics may stimulate phage infection, changing the phenotype of the target host, and phages may interfere with drug-resistant mechanisms, making bacteria more vulnerable (Comeau et al., 2007; Chan et al., 2016; Dickey and Perrot, 2019; Chegini et al., 2021). In many cases, PAS is necessary for successful therapy due to its better ability to degrade biofilm complex. At the same time, more than one factor is applied and all of them present different mechanisms of action (Roszak et al., 2022). Phages are considered more effective against biofilm due to the production of polysaccharide depolymerases which loosen matrix structure and help antibiotics reach the bacteria cell surface. Another mode of action is the lysis of cell from exterior parts of biofilm which results in uncovering the deeper layers of cells, and giving them access to nutrients and oxygen. This makes bacteria more metabolically active and more susceptible to an antibiotic (Park et al., 2017). The effectiveness of such therapy also depends on the dosage of antibiotics and phages, time and order of administration, adsorption rate, burst size, latent period, and external physical factors such as pH and temperature (Morrisette et al., 2019).

**Table 2:** Examples of phage-antibiotic synergy therapy in combating polymicrobial biofilm in *in vitro* studies.

No.	Pathogens	Aim of the study	Phages used	Antibiotic used	Experimental model	Outcome	References
1.	*P. aeruginosa* PAO1/ *S. aureus* ATCC 25923	Dual-species biofilm eradication	Phage (EPA1) against *P. aeruginosa*	Gentamicin	biofilm formation in 24-well plates for 48 h at 37°C with shaking, Both bacteria 10^8^ CFU/mL. Then one of the antibiotic concentrations (MIC or 8xMIC) and phage at MOI = 1 were added simultaneously for 24 h treatment or the second agent was added after 6 h	In the control, *P. aeruginosa* and *S. aureus* cells, concentration was 1.4 × 10^9^ CFU/mL and 2.3 × 10^5^ CFU/mL, respectively Gentamicin only (1 × MIC (4 mg/L) and 8 × MIC) reduced 3.3 orders-of-magnitude and 4.6 orders-of-magnitude of *P. aeruginosa* cells, respectively. Phage treatment reduced by 0.7 orders of magnitude of *P. aeruginosa* cells. None of the individual treatments showed an impact on the *S. aureus* population The simultaneous treatments: phage-gentamicin 1 × MIC resulted in 4.1-orders-of-magnitude reduction of *P. aeruginosa* and 0.4 of *S. aureus*, phage-gentamicin 8 × MIC resulted in 4.6-orders-of-magnitude reduction of *P. aeruginosa* and 0.8 of *S. aureus* Preliminary phage treatment (6 h) before gentamicin 1 × MIC reduced 6.3 orders-of-magnitude the *P. aeruginosa* population and had no impact on the *S. aureus* population. Phage-gentamicin 8 × MIC almost eradicated *P. aeruginosa* cells (approx. 7 orders-of-magnitude reduction) and reduced 2-orders-of-magnitude *S. aureus* population	Akturk et al. (2019)
2.	*P. aeruginosa* PAO1/*S. aureus* ATCC 25923	Dual-species biofilm eradication	Phage SAFA against *S. aureus* and phage EPA1 against *P. aeruginosa*	Gentamicin	biofilm formation in 24-well plates for 48 h at 37°C, with shaking Both bacteria inoculum 10^8^ CFU/mL. Then treatment (various combinations of phages and gentamicin) biofilm formation on wound model - biofilm was treated with the antimicrobials (GEN 4 mg/L, phages MOI = 1); alone, in simultaneous (EPA1 + SAFA+GEN) or sequential combinations (first EPA1 + SAFA and then GEN with 6 h delay), then incubation at 37°C for 24 h	Single-dose, wound model 6 h treatment: Phage EPA1 treatment reduced the *P. aeruginosa* population by 1.5 log, phage SAFA did not reduce the *S. aureus* population, treatment with GEN reduced the *P. aeruginosa* population by 1.0 log and *S. aureus* by 0.9 log 24 h treatment: Phage EPA1 treatment reduced the *P. aeruginosa* population by 1.5 log, phage SAFA did not reduce the *S. aureus* population, treatment with GEN reduced the *P. aeruginosa* population by 3.4 log and *S. aureus* by 1.7 log When EPA1 + SAFA, followed by GEN 6 h later were applied, biofilm reductions of 4.8 and 2.3 log were observed for *P. aeruginosa* and *S. aureus*, respectively Multiple doses, 24 well plate A single dose of phages and GEN, phages alone, and GEN alone for 8 h resulted in a reduction of *P. aeruginosa* population by 0.8, 1.1, and 1.3 log, and *S. aureus* populations by 0.2, 0.8, and 1.0 log. The second dose led to biofilm reductions ranging from 1.1 to 5.0 log for *P. aeruginosa* and 1.6 to 6.8 log for *S. aureus* The most effective reduction was obtained following multiple doses of EPA1 + SAFA+GEN, with a 6.2 log reduction for *P. aeruginosa* and 5.7 log for *S. aureus*	Akturk et al. (2023)
3.	*S. aureus* MRSA ATCC 37741/*S. epidermidis* ATCC 12228	Dual-species biofilm eradication	Phage type 92 (ATCC 33741-B) against *S. aureus*	Teicoplanin	biofilm formation in 96-well plates for 48 h at 37°C with shaking, then phage at MOI = 10 or teicoplanin (10 mg/L) or both agents treatment for 12 h	In untreated mixed-culture biofilms, MRSA outcompeted *S. epidermidis* The most effective treatment was phage alone: *S. aureus* reduction approx. 1.5 Log_10_ CFU/cm^2^, *S. epidermidis* approx. 0.25 Log_10_ CFU/cm^2^; teicoplanin alone: *S. aureus* reduction approx. 0.4 Log_10_ CFU/cm^2^, *S. epidermidis* no reduction; combined therapy: *S. aureus* reduction approx. 1.05 Log_10_ CFU/cm^2^, *S. epidermidis* no reduction (data read from the original figure) *S. epidermidis* acquired increased tolerance to teicoplanin	Infect et al. (2016)
4.	*P. aeruginosa* PA01/ *C. albicans* C11	Dual-species biofilm eradication	Phage Motto (NCBI accession number ON843697) against *P. aeruginosa*	Fluconazole, cefotaxime, ciprofloxacin, gentamicin, meropenem and tetracycline	biofilm formation in 96-well plates for 6 or 24 h at 37°C both microorganisms (10^5^ CFU/mL), then phage (10^2^ to 10^9^ or 10^12^ PFU/mL) and fluconazole (2 to 128 mg/L) or cefotaxime, ciprofloxacin, gentamicin, meropenem, and tetracycline (0.5 to 128 mg/L) were added, incubation for 16 h	The eradication of biofilm was impossible in the presence of phage alone or antibiotics alone High phage and fluconazole concentrations reduced biofilm up to 30%, with 6 and 24 h biofilm samples, but full eradication was not observed Phage had a positive impact on the removal of the dual-species biofilm in combination with the exposure to fluconazole Even at the highest concentration of cefotaxime, ciprofloxacin, gentamicin, meropenem or tetracycline and highest phage titer tested, biofilms remained unaltered	Manohar et al. (2022)
5.	*S. aureus* ATCC 6538/*C. albicans* ATCC 10231	Elimination of *S. aureus* from dual-species biofilm	Phages vB_SauM-A and vB_SauM-D against *S. aureus*	Ciprofloxacin	biofilm formation in 96-well plates for 24 h at 37°C, then phages (10^7^ PFU/mL) and ciprofloxacin (1 to 32 mg/L) were added separately or together	The individual treatments with phage A or D or both lead to 50% reduction of biofilm specific activity and 67% reduction of *S. aureus* population; individual treatment with ciprofloxacin lead to 83–23% reduction of biofilm specific activity depending on concentration (32–1 mg/L) and 55% reduction of *S. aureus* population (ciprofloxacin 1 mg/L) The combined treatment: the reduction of biofilm specific activity was 82 to 69% depending on ciprofloxacin concentration (32–1 mg/L) and 95% reduction of *S. aureus* population (ciprofloxacin 1 mg/L) Presence of *C. albicans* lead to less *S. aureus* reduction in comparison to mono-species biofilm	Roszak et al. (2022)
6.	*P. aeruginosa* ATCC 27853/*S. aureus* (MRSA) ATCC 43300	Dual-species biofilm eradication	Phages Sb-1 and PYO	Ciprofloxacin	biofilm formation on porous sintered glass bead for 24 h at 37°C, 5 × 10^6^ CFU/mL *S. aureus*, 5 × 10^3^ CFU/mL *P. aeruginosa*; then phage treatment: simultaneously addition of PYO or Sb-1 + PYO or Sb-1 + PYO + sub-inhibitory concentration of ciprofloxacin; staggered exposure to PYO or PYO + Sb-1 for 3, 6, 12, or 24 h followed by a 24 h-exposure to sub-inhibitory concentrations of ciprofloxacin	Delay on the heat production was observed when PYO was applied, and it was enhanced when Sb1 was added, no complete inhibition of the biofilm was observed A reduction of more than 2 log_10_ of MRSA and 1 log_10_ of *P. aeruginosa* cells was observed after exposure to PYO The combination of PYO + Sb-1 showed a complete eradication of MRSA cells and no substantial reduction of *P. aeruginosa* cells PYO+ ciprofloxacin 16–64 mg/L decreased heat flow production reduced over 90% PYO + Sb-1 + ciprofloxacin 4 mg/L reduce over a 90% of the heat flow production The highest anti-biofilm activity was observed when the antibiotic (2 mg/L or 1 mg/L) was added after 12 h of pre-exposure to either PYO or PYO + Sb-1, no presence of bacteria on the beads was observed	Tkhilaishvili et al. (2020)

Another approach is to combine phages with nanoparticles. That solution may enhance phage penetration through biofilm. Moreover, nanoparticle migration in matrix might be modulated by a magnetic field in *ex vivo* models. Li et al. (2017) investigated how polyvalent phages (PEL1) immobilized onto Fe_3_O_4_-based magnetic colloidal nanoparticle clusters (CNC) coated with chitosan (PEL1-CS-Fe3O4) penetrate *P. aeruginosa/E. coli* dual-species biofilm. The complex penetration was facilitated under a small magnetic field (660 gauss), leading to better plaque formation capability of PEL1 and removal of 88.7 ± 2.8% of the biofilm formed on a glass surface after 6 h of treatment. The usage of such a particle complex physically disrupts the biofilm and mitigates phage dilution, which, in turn, allow to keep a high concentration of phages and facilitate phage tail fibers exposition to the hosts (Li et al., 2017). Another study where phages were covalently conjugated with magnetic CNCs shows that this approach is noteworthy. Yu et al. (2019) used phages PEB1 or PEB2 conjugated with CNCs of different sizes to combat *P. aeruginosa/ E. coli* dual-species biofilm and *P. aeruginosa/E. coli/B. subtilis* and *Shewanella oneidensis* multi-species biofilm. Smaller complexes disrupted the biofilm bottom layer and detached the biofilm within 6 h with efficiency of 98.3 ± 1.4% for dual-species biofilm and 92.2 ± 3.1% for multi-species biofilm. Larger complexes were less effective, implying that the size of nano-phage complex matters (Yu et al., 2019). It was reported that magnetic field might influence bacteriophage development. Phages T4 for *E. coli* and vB_SauM_A for *S. aureus* exposed to a rotating magnetic field enhance their adsorption and propagation rate (Struk et al., 2017; Konopacki et al., 2020; Grygorcewicz et al., 2022). In addition, a magnetic field might modulate the metabolism of bacteria and other microorganisms (Jabłońska et al., 2022).

In addition to antibiotics and nanoparticles, other chemical compounds or groups of compounds might be combined with phages to minimize formation and eradicate polymicrobial biofilm. Chhibber et al. (2015) tested how bacteriophages combined with xylitol will eradicate *K. pneumoniae/P. aeruginosa* dual-species biofilm formed on polycarbonate disks. *K. pneumoniae*-specific depolymerase-producing phage KPO1K2 and *P. aeruginosa* specific non-depolymerase-producing phage Pa29 led to 2.13 and 1.27 log_10_ CFU/mL reduction of *K. pneumoniae* and *P. aeruginosa* cell counts, respectively in 1-day-old biofilm. They obtained slightly worse results for 2-day-old biofilm. The authors emphasize that depolymerase-producing phage was crucial for matrix disruption. The addition of xylitol to the system significantly enhanced the antibiofilm activity of phages and caused complete elimination of *K. pneumoniae* both in 1- and 2-day-old biofilms and also 3.5 and 3.02 log_10_ CFU/mL reduction of *P. aeruginosa* in 1- and 2-day-old biofilm, respectively. Xylitol may diffuse into the biofilm and accumulate as a toxic, non-metabolizable sugar alcohol phosphate, thus inhibiting bacterial growth, or it can hinder stress proteins that arise in the biofilm (Ichikawa et al., 2008; Chhibber et al., 2015). An interesting approach was presented by Oliveira et al. (2018), who used chestnut honey bacteriophages (vB_EcoS_CEB_EC3a and vB_PaeP_PAO1-D) against *P. aeruginosa/E. coli* dual-species biofilm formed on polystyrene and porcine skin. Honey has antimicrobial properties associated with high osmolarity, low availability of water, hydrogen peroxide production, acidic pH level, and the presence of methylglyoxal. The results of using different combinations of phage and honey showed that *E. coli* cell number reduction in biofilm depends on the applied treatment time and honey concentration. In the case of *P. aeruginosa,* combined treatment brought better results than phage or honey alone, however, without presenting a synergy effect on the polystyrene model. *E. coli* elimination from dual-species biofilm formed on porcine skin model was the most effective using phage and 50% honey and led to 1.4 log reduction at 24 h post-treatment. The combination of phage and honey acts synergistically in *P. aeruginosa* cell elimination at both concentrations (25 and 50%), leading to 2.2 log_10_ and 2.3 log_10_ higher cell reduction than the sum of phage and honey alone (Oliveira et al., 2018).

Phage-delivered enzymes are the next option to eliminate polymicrobial biofilm. Their main advantages are host specificity and easy matrix penetration and removal. Skillman and Sutherland (1999) proposed the usage of polysaccharide depolymerases isolated from a bacteriophage infecting *E. agglomerans* to degrade EPS in a dual-species biofilm formed with *K. pneumoniae*. Such treatment caused limited adhesion of *E. agglomerans* to *K. pneumoniae*, degradation of EPS, and effective removal of both species from the surface, even though the used enzyme was specific toward *E. agglomerans* only. This effect might have been caused by the proximity of both species or the larger contribution of *E. agglomerans* EPS in the mixed biofilm. Schuch et al. (2017) used bacteriophage lysin *CF*-301 and combined it with lysostaphin to target *S. aureus* and *S. epidermidis* in mixed biofilm formed on various surfaces (polystyrene, surgical mesh, and catheters). Dual-species biofilm was susceptible to disruption by *CF*-301 applied at concentrations down to 0.032 mg/L over 24 h. The reduction of both species on catheter and surgical mesh reached over 90% and over 80% on 24-well polystyrene plates. The good enzymatic activity against both species is reasonable because they belong to the same genus. However, more than one protein should be used when more phylogenetically distant species form a mixed biofilm. This approach was investigated by Manoharadas et al. (2023), who used two engineered enzybiotics (BP404 5 mg/L and P16-17/100 5 mg/L) against a dual-species biofilm formed by *S. aureus* and *E. faecalis* in an inert glass surface. The chimeric protein P16-17/100 was constructed, linking domains from endolysin P16 and minor tail protein P17 from phage φ44AHJD. Protein cocktail usage resulted in significant biofilm dispersal (absorbance OD575 reduction from 0.7 to less than 0.1) and more than 90% reduction of both species cells embedded in the matrix after 16 h of treatment.

Bacterial vaginosis (BV) is a common vaginal infection caused by anaerobic pathogens such as *Gardnerella vaginalis*, *Fannyhessea vaginae*, and *Prevotella bivia*, usually forming a polymicrobial biofilm. Therapy of BV usually relies on metronidazole and clindamycin treatment. However, sometimes, these antibiotics do not lead to the complete eradication of pathogens. The curation of biofilm-associated BV is challenging. Therefore, Landlinger et al. (2021) generated engineered endolysin Pm-477 encoded on *Gardnerella* prophages as an alternative treatment. The endolysin actively killed *G. vaginalis* in mono- and dual-species communities with *Lactobacillus crispatus*. Moreover, the efficacy of PM-477 was tested by fluorescence *in situ* hybridization on vaginal samples of 15 women with BV. Endolysin eliminated *Gardnerella* bacteria in 13 cases and physically dissolved the biofilm matrix. The remaining vaginal microbiome remained unaltered. Castro et al. (2022) also tested previously synthesized engineered phage endolysin PM-477 to disrupt dual-species biofilms composed of *G. vaginalis/F. vaginae* or *G. vaginalis/P. bivia* in *in vitro* study. In all dual-species biofilms, endolysin prevented biomass accumulation (from 24 to 48 h) but did not reduce existing ones. In *G. vaginalis/F. vaginae,* biofilm cell viability reduction was not obtained, but in *G. vaginalis/P. bivia,* biofilm reduction reached a 3 log_10_ CFU. The phage endolysin had high anti-*G. vaginalis* and slightly anti-*P. bivia* but no anti-*F. vaginae* activity.

Johnston et al. (2023) also investigated how endolysin therapy against *G. vaginalis* biofilm works *in vitro.* In their study, a four-species biofilm made of *G. vaginalis, F. vaginae, P. bivia*, and *Mobiluncus curtisii* was treated using an anti-*Gardnerella* endolysin (CCB7.1) as this species is the most abundant in polymicrobial community. The reduction of live cells of *G. vaginalis* reached 1–2 log_10_ after 24 h of endolysin treatment in all tested concentrations (128, 256, and 512 μg/mL) and a slight reduction of *M. curtisii* when the highest concentration of endolysin was applied. Worth mentioning is that CCB7.1 was ineffective against commensal lactobacilli. Novel endolysins against *G. vaginalis* are still being searched. Arroyo-Moreno et al. (2022) identified 84 diverse anti-*Gardnerella* endolysins and selected 5 (CCB2M94_8, CCB7.1, CCB8.1, CCB2.2, and CCB4.1) with the best properties. All of them could disturb *G. vaginalis/Atopobium vaginae* dual-species biofilm in the concentration of 200 μg/mL and had no activity against commensal lactobacilli.

## Bacteriophages in the fight against chronic infections

*In vitro* studies provide valuable data about phage therapy efficiency against polymicrobial biofilms. Research shows that phages themselves or in combination with antibiotics or other substances can reduce biofilm formed on various surfaces, e.g., polystyrene, glass, stainless steel, or silicone (urine catheters) (Curtin and Donlan, 2006; Carson et al., 2010; Kaźmierczak et al., 2022). Promising results from *in vitro* studies allowed to start more comprehensive clinical trials using bacteriophages. Even though in some countries (Georgia, Russia, Poland) phage therapy has been used for many years, the Western world has only recently started the first attempts to treat patients with phages. Reported cases when phages or their enzymes were included in therapy refer to wound infections, bone infections, surgical site infections, etc.

Bone and joint infections are the hardest to cure and are usually related to post-traumatic or implant infections. Bacteria quickly form polymicrobial biofilm and can persist in osteoblasts or synovial cells, implicated in chronicity and recurrence, usually requiring heavy surgery with implant exchange. Bacteria mainly isolated from bone infections are *S. aureus*, coagulase-negative staphylococci, *Cutibacterium acnes*, *Streptococcus* spp., *Enterobacteriaceae*, and *P. aeruginosa* (Ferry et al., 2021). When antibiotics fail, phage therapy is proposed to patients.

Nir-Paz et al. (2019) successfully treated a 42-year-old male patient with a trauma-related left tibial infection caused by extensively drug-resistant *Acinetobacter baumannii* and multidrug-resistant *K. pneumoniae.* Patient with trauma was first treated with external fixation, irrigation, and debridement, plus left leg fasciotomies and a prolonged course of antibiotics: 6 weeks of piperacillin/tazobactam, initially followed by an 8-week course of meropenem and colistin. After 7 months of unsuccessful therapy, phages were included. The patient received a phage cocktail (ɸAbKT21phi3 and ɸKpKT21phi1 in concentration 5 × 10^7^ PFU/ml each), colistin (4.5 × 10^6^ units/bid), and meropenem intravenously. The first effect of curation was visible after a few days, and 8 months post-treatment, no bacteria were detected. Phage-antibiotic therapy saved the patient’s leg from amputation. Onsea et al. (2019) provide further instances of successful phage therapy. The group developed a protocol for intraoperative phage application and postoperative use of a draining system. They reported three successful curation of patients with polymicrobial bone infection: Patient 1 (infection: the trauma of pelvis; bacteria: *P. aeruginosa, S. epidermidis*; antibiotics used beside phages: for 3 months, vancomycin, rifampicin, moxifloxacin; phage therapy: for 7 days, BFC1 phage cocktail contains phages against *S. aureus* and *P. aeruginosa* 10^7^ PFU/mL); patient 2 (infection: the trauma of femur; bacteria: *P. aeruginosa, S. epidermidis*; antibiotics used in addition to phages: for 6 weeks, vancomycin, colistin, fosfomycin; phage therapy: for 10 days, BFC1 phage cocktail); patient 3 (infection: trauma of femur; bacteria: *S. agalactiae, S. aureus*; antibiotics used in addition to phages: for 3 months, vancomycin, clindamycin, moxifloxacin; phage therapy: for 9 days, BFC1 phage cocktail). After 8 or 16 months, no signs of infection were observed (patients 1 and 3), and patient 2 needed further treatment. Van Nieuwenhuyse et al. (2021) report the case of a 13-year-old patient who developed chronic polymicrobial biofilm infection of a pelvic bone allograft. *Clostridium hathewayi*, *P. mirabilis*, *Finegoldia magna,* and methicillin-susceptible *S. aureus* were isolated from the infectious site. Conventional therapy (intravenous antibiotics and surgical debridement) with anti-*S. aureus* phage treatment (BFC1 phage cocktail) *in situ* was implemented. At first, therapy led to marked clinical and microbiological improvement, but it failed to prevent a recurrence of infection later.

Difficult to treat and chronic bacterial infections can occur at different sites of infection. They are born by various bacteria that cannot be eliminated with antibiotic therapy due to the formation of polymicrobial biofilm and the possibility of cell survival (Morozova et al., 2018). Phage therapy was proposed in many cases, referring to polymicrobial infections. Püschel et al. (2022) reported a case of successful treatment of drive line infection acquired after left ventricular assist device (LVAD) implantation with a combination of antibiotics, debridement, and local bacteriophage treatment. *P. mirabilis* and *S. aureus* were isolated due to unsuccessfully treated surgically for a driveline phage therapy was used. Phage cocktail containing phages against *E. coli, S. aureus, P. aeruginosa, S. pyogenes, P. vulgaris,* and *P. mirabilis* (10^7^ PFU) was applied to the site of infection. The wound was healing well; the patient received cotrimoxazole for 20 days. Afterward, only *S. aureus* was detected in the infection site, and further flucloxacillin treatment was applied. In a follow-up examination 8 months later, the primary site of infection was free from bacteria.

Another example might be considered a success. However, the patient died long after phage therapy. Rubalskii et al. (2020). present a case of 52-year-old patients with a prosthetic infection after aortic arch replacement. Implant drainage and bronchial lavage were infected with *S. aureus, E. faecium. P. aeruginosa,* and *E. faecium*. Following the ineffectiveness of antibiotic therapy, a combination of phages (10^8^ PFU/mL of *Staphylococcus* phage CH1, *Enterococcus* phage Enf1, *Pseudomonas* phage PA5, and *Pseudomonas* phage PA10), was applied in combination with two applications of gentamicin and daptomycin locally during the intraoperative phase, and a long-term intravenous application of cefepime, daptomycin, linezolid, and tobramycin was employed. After the intervention, *S. aureus, E. faecium*, and *P. aeruginosa* were undetected. However, the patient died after 2 months due to a new bacterial infection.

A research group from Eliava Phage Therapy Center, Tbilisi, Georgia (Nadareishvili et al., 2020) presents cases of successful phage treatment of polymicrobial infection related to biofilm (Nadareishvili et al., 2020). Patient 1, a 69-year-old male patient with a diabetic foot ulcer, was infected with following bacteria: *Burkholderia cepacia, S. aureus,* and *E. faecalis.* The staphylococcus phage and Intesti bacteriophage cocktail (consisting of *Shigella* spp., *Salmonella* spp., *E. coli, Proteus* spp., *S. aureus*, *P. aeruginosa,* and *E. faecalis* phages) were applied daily in the site of infection and orally for 40 days. The size of the wound was reduced after a few weeks; in addition, there was no recurrence after 1 year of treatment. Patient 2, a 68-year-old male patient with a postsurgical infection (after skin graft surgery), had two infections: the first infection was mono-species, and the second one was caused by *S. aureus* and *Serratia marcescens*. After the application of staphylococcus phage daily at the site of infection and orally for 3 months, the infection was resolved, and the tissue healed completely. Another example is reported in cooperation with Johri et al. (2021) group. Patient with chronic bacterial prostatitis (CBP) infected by methicillin-resistant *S. aureus* (MRSA), *Staphylococcus haemolyticus, E. faecalis, and Streptococcus mitis* was first unsuccessfully treated with antibiotics. Then, Pyo (a cocktail of phages against *Streptococcus* spp., *Staphylococcus* spp., *E. coli*, *P. aeruginosa*, and *Proteus* spp.) and Intesti bacteriophage cocktail, combined with additional *Staphylococcal* phage, was introduced in three forms: oral liquid, rectal suppositories, and urethral installations. After 5 days of therapy, the patient’s body temperature normalized. The therapy was prolonged, and isolated from patient bacteriophage against *S. mitis* was included. After almost a year, in a follow-up examination, semen and expressed prostatic secretion were free from bacteria, and the prostate was small and firm by rectal palpation.

## Conclusion and perspectives

In conclusion, bacteria and other microorganisms prefer to organize themselves in multi-species communities. Such biofilms are difficult to cure using antibiotic therapy and to remove from abiotic surfaces. Due to the persistence of multi-species biofilms, alternative methods of their eradication are being developed. Bacteriophages are one of the solutions. Studies have been conducted using both wild-type and genetically modified or polyvalent phages. In addition, they can be successfully used in combination with antibiotics or other chemical molecules. A separate group consists of enzymes and modified enzymes produced by phages. All these methods allow for better penetration of the biofilm matrix and reaching the surface of the target bacterial strains. The use of phage therapy is also increasingly used in medicine in the treatment of severe multi-species infections. However, the routine use of bacteriophages in medicine still requires a lot of research, including optimization and legislative work. However, despite further work required, bacteriophages and therapies using them to any extent are the future in treating bacterial infections. These viruses are and will be increasingly used to prevent bacteria in the hospital environment and other cases, e.g., in the food industry, veterinary medicine, or agriculture.

## Author contributions

MG: Conceptualization, Data curation, Funding acquisition, Methodology, Project administration, Validation, Visualization, Writing – original draft, Writing – review & editing. DM: Writing – original draft. PO: Writing – original draft. AC: Writing – original draft. NS: Writing – original draft. EC-H: Writing – original draft. BD: Writing – original draft. BG: Conceptualization, Formal analysis, Funding acquisition, Resources, Supervision, Validation, Visualization, Writing – original draft, Writing – review & editing.
